# Supplementary material for: The Hippo terminal effector YAP boosts enterovirus replication in type 1 diabetes
Source: Nat Commun. 2025 Oct 6;16:8882. doi: 10.1038/s41467-025-64508-6 (PMC12500894; doi:10.1038/s41467-025-64508-6)
Supplement: Supplementary file 1 — Supplementary Information [file 41467_2025_64508_MOESM1_ESM.pdf]

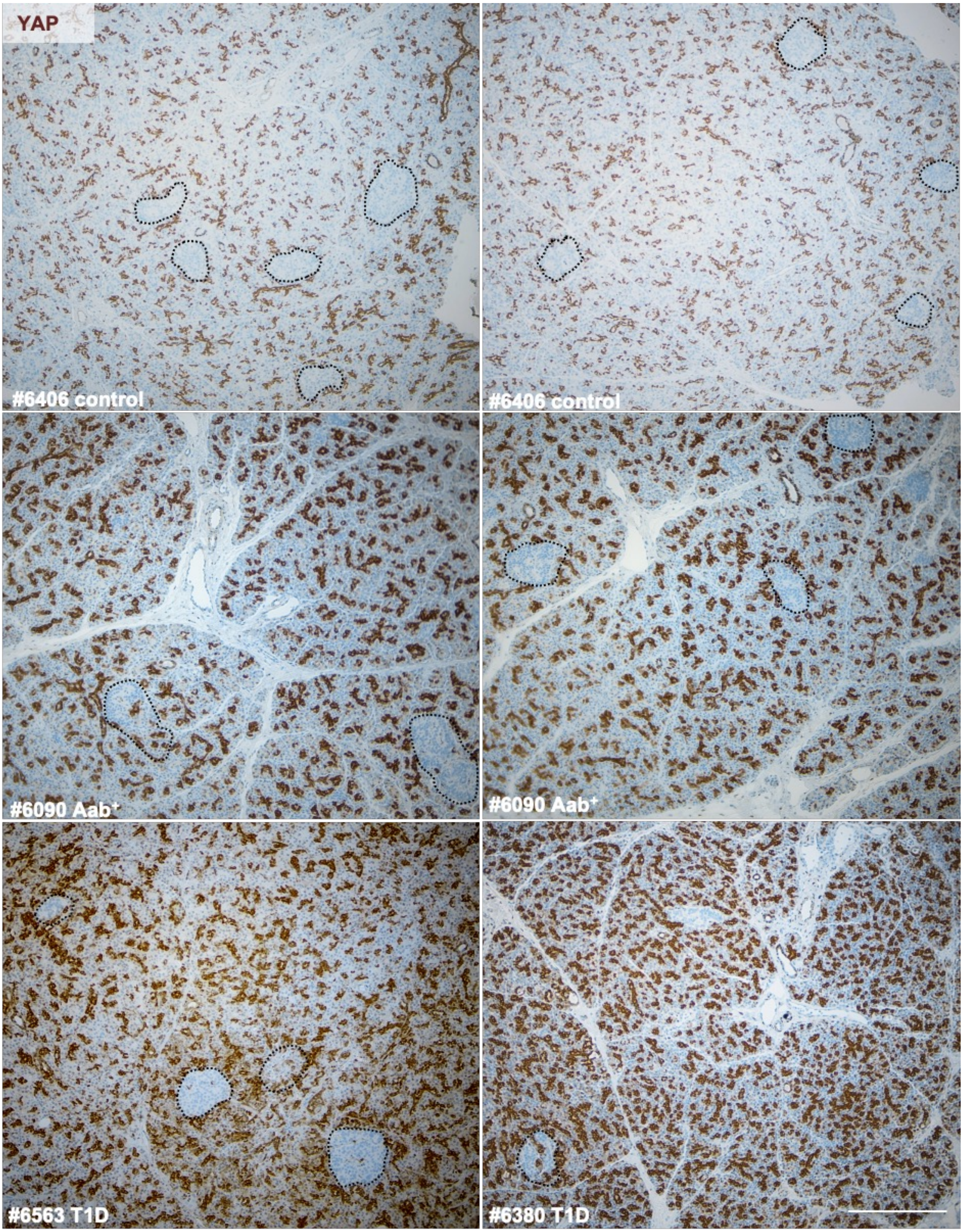

Figure S1. YAP is highly upregulated in the pancreas of T1D and AAb⁺ organ donors. YAP protein labeling of FFPE sections of pancreases from control, AAb⁺ organ donors without diabetes and donors with T1D from the nPOD pancreas collection; larger views of pancreases from Figure 1A. Scale bar depicts 100μm.

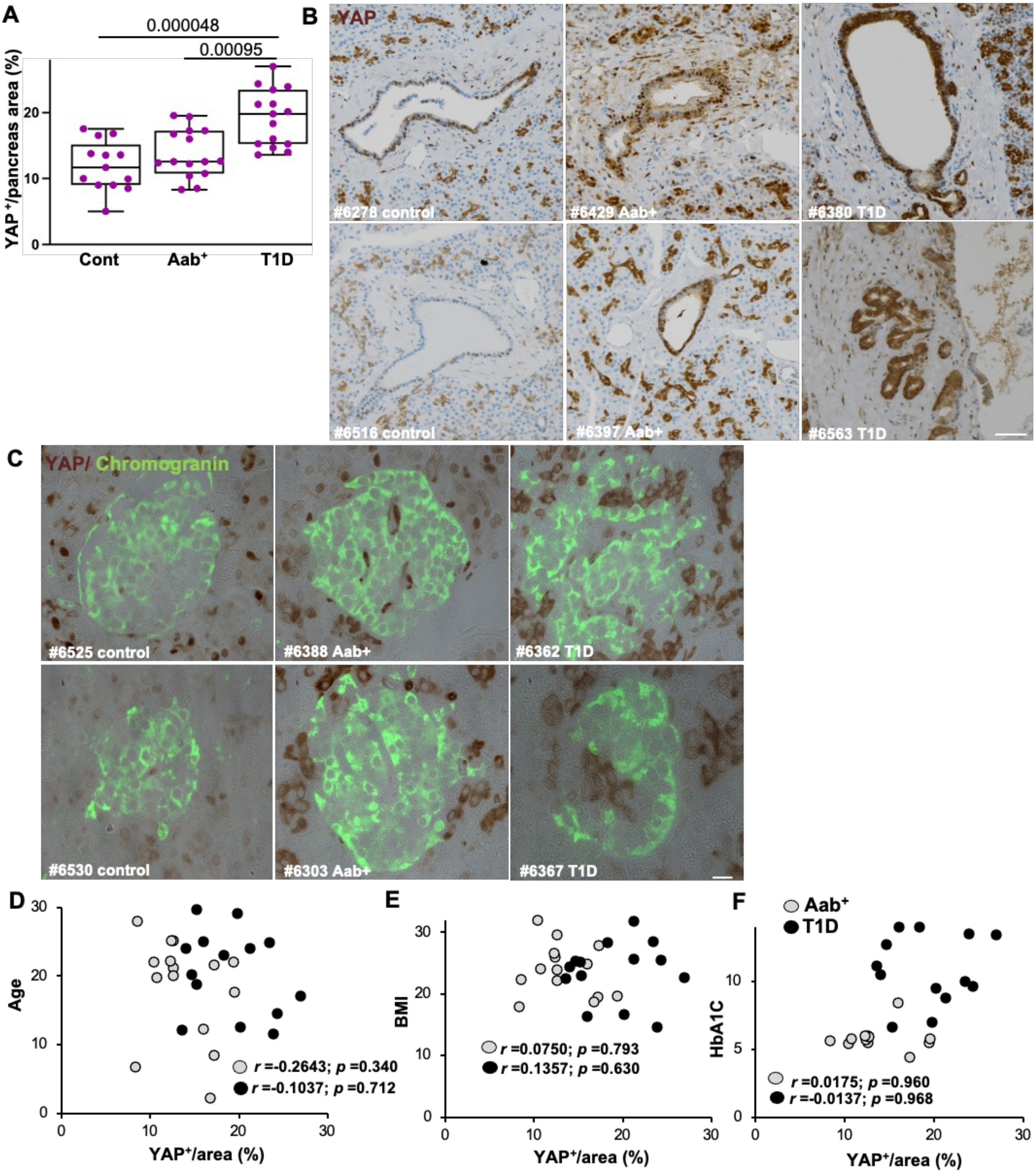

Figure S2. YAP is elevated in pancreases of T1D and AAb⁺ organ donors. (A) Quantification of the percentage of YAP⁺ area in the exocrine pancreas of control donors without diabetes (n=13), AAb⁺ (n=15), and T1D donors (n=15; related to Fig.1A,B). **(B,C)** Representative images of **(B)** YAP in the exocrine pancreas and **(C)** of YAP (brown) and chromogranin (green) in the islet area. **(D-F)** Association between YAP protein expression in pancreases and **(D)** age, **(E)** BMI and **(F)** with HbA1C in AAb⁺ and T1D. Box plot showing single analytes and median (box and whiskers; min to max show all points). Scale bars depict 50μm (B) and 10μm (C). Data are expressed as means ± SEM. *P*-values were calculated by one-way ANOVA with Holm-Sidak multiple comparisons correction.

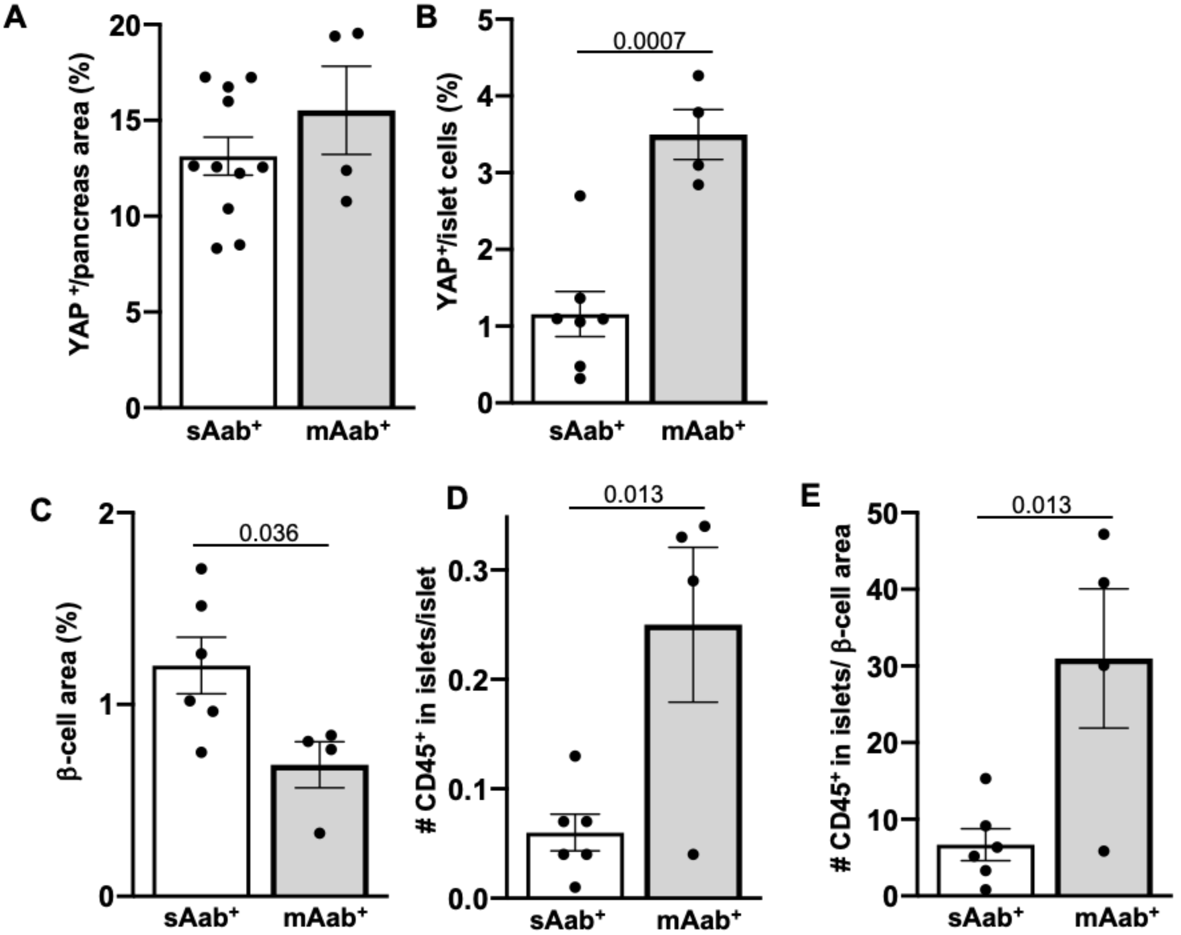

Figure S3. Sub-cluster analyses of AAb⁺ pancreata. %YAP protein-positive area throughout the pancreas **(A)** and within chromogranin⁺ islet areas **(B)** from 11 **(A)** or 7 **(B)** single AAb⁺ donors and four multiple AAb⁺ donors (three double AAb⁺⁺ and one triple AAb⁺⁺⁺). **(C)** β-cell area (insulin⁺ are shown as % of whole pancreas section area). **(D,E)** number of CD45⁺ immune cells within islets divided by **(D)** the number of islets or **(E)** by the whole β-cell area. C-E from 6 single AAb⁺ and four multiple AAb⁺ donors. Data are expressed as means ± SEM. Data from donors in (C-E) were partially re-analysed from our previous study (Geravandi et al, Cell Rep Med, 2021). *P*-values were calculated by two-tailed unpaired Student *t*-test.

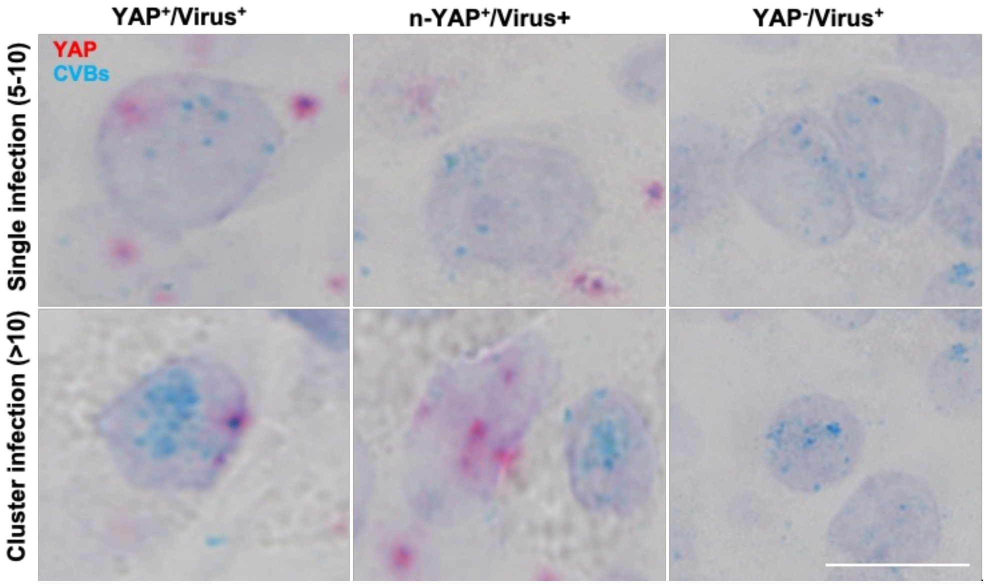

Figure S4. Various categories of infected pancreatic cells. Representative images of single (upper panel; 5-10 puncta of CVB-RNA expression/cell) and cluster (lower panel, >10 puncta of CVB-RNA expression/cell) infections of three different categories of YAP-viral RNA double positive cells (YAP^+/CVB^+), CVB-positive cells in close proximity of YAP-positive neighbor cells (n-YAP^+/CVB^+) or YAP-negative but CVB-RNA-positive cells (YAP^-/CVB^+) in human pancreases from AAb^+ and T1D donors. Scale bar depicts 10μm.

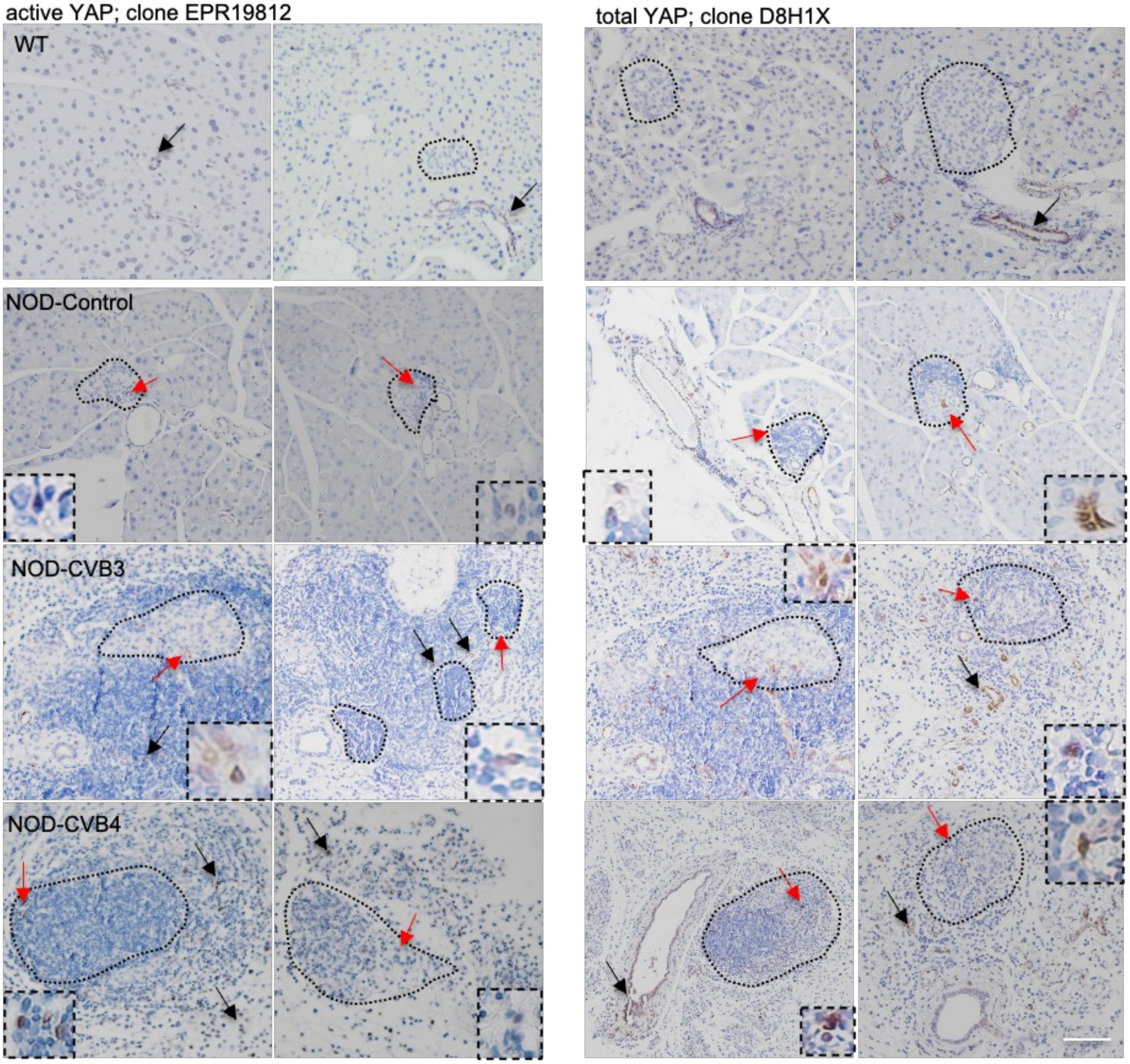

Figure S5. YAP is increased in the pancreas of CVB infected diabetic NOD mice. Representative stainings of active YAP (non-phosphorylated form) and total YAP in brown and haematoxylin in blue in FFPE pancreas sections of WT C57Bl/6J and diabetic NOD mice. Islet are marked by dotted lines. Normoglycemic age- and sex-matched non-obese diabetic (NOD) mice at 11-12 weeks old were injected intraperitoneally with either 400 plaque-forming units (pfu) CVB3 or CVB4 or DMEM vehicle and pancreata dissected 1-week post-infection. Larger magnifications show YAP^{+} cells in the islets (red arrow), black arrow points to YAP^{+} cells in the exocrine pancreas. Scale bar depicts 100µm.

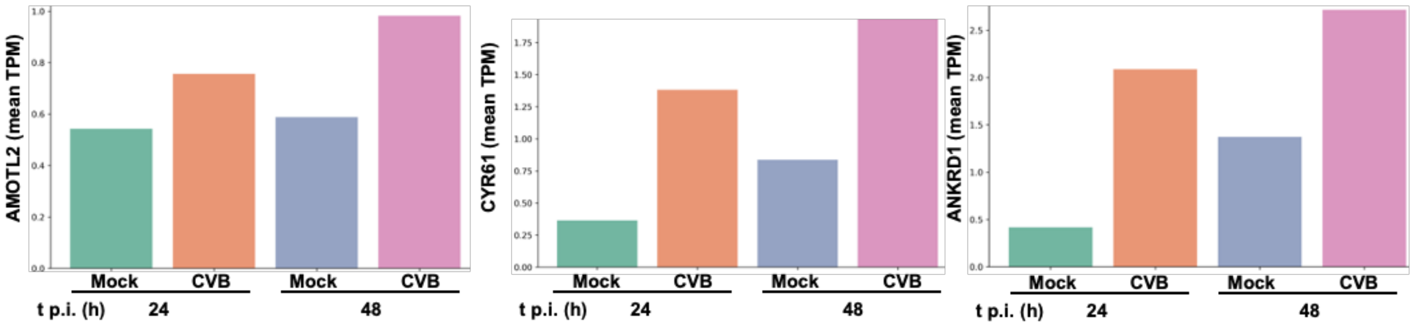

Figure S6. YAP target genes are increased in CVB-infected β-cells. Bar plot showing the expression levels of *AMOTL2, CYR61, and ANKRD1* in human stem cell-derived β (SC-β) cells infected with CVB4 at different time points. Data were reanalyzed from a publicly available bulk RNA-seq dataset.

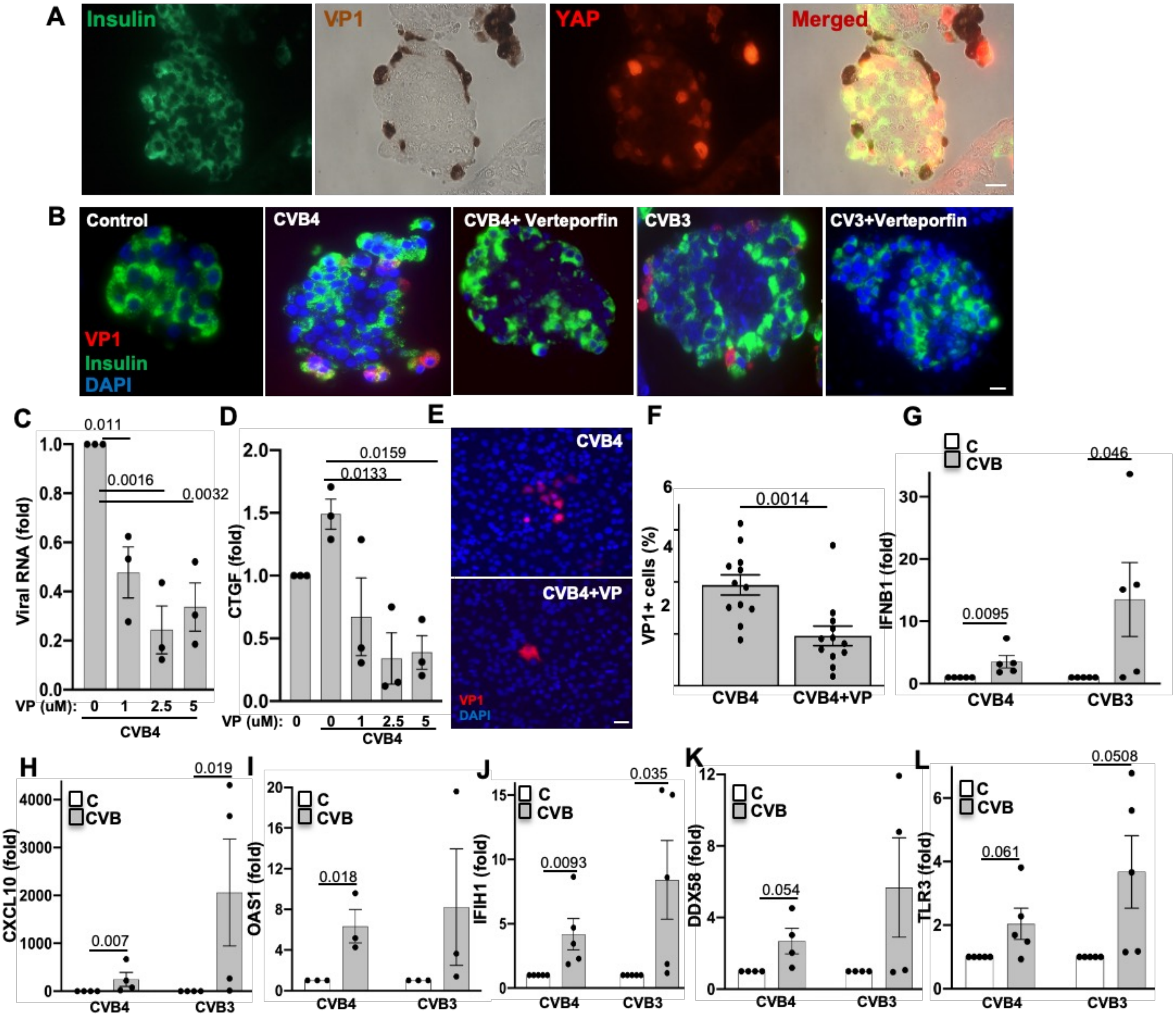

Figure S7. YAP regulates coxsackieviruses replication and islet inflammation. (A) Representative image of triple VP1-, YAP-, and insulin-positive β-cells in human islets transduced with Ad-YAP or Ad-LacZ control and then infected with CVB4 (MOI=10) for 48h (n=4 organ donors). **(B)** Representative image of triple VP1-, DAPI and insulin-positive β-cells of human islets (50%) co-cultured with exocrine cells infected with CVB3 and -4 and treated with or without 2.5 uM verteporfin (VP) for the last 24h. Quantitative percentage of %VP1/insulin^{+} cells is shown in Fig.6I. **(C-F)** PANC-1 cells infected with CVB4 for 48h treated with or without 0-5μM VP for last 24h. **(C)** Intracellular CVB4 RNA genome of PANC1 cells (n=3 independent experiments). **(D)** qPCR for *CTGF* mRNA expression in PANC1 cells (n=3 independent experiments). **(E,F)** Representative images **(E)** and quantitative percentage of VP1-positive cells **(F)** are shown (n=12 independent positions). **(G-L)** Human islets infected with CVB3 and -4 (MOI=10) for 48h. qPCR for *IFNB1*, *CXCL10*, *OAS1*, *IFIH1*, *DDX58* and *TLR3* mRNA expression in isolated human islets normalized to actin (G,J,L: n=5, H,K: n=4, I: n=3 organ donors). Data are expressed as means ± SEM. *P*-values were calculated by one-way ANOVA with Holm-Sidak multiple comparisons correction for C and D, by two-tailed unpaired Student *t*-test for F and by two-tailed ratio paired Student *t*-test (G-L). Scale bars depict 10μm (A,B) and 5μm (E).

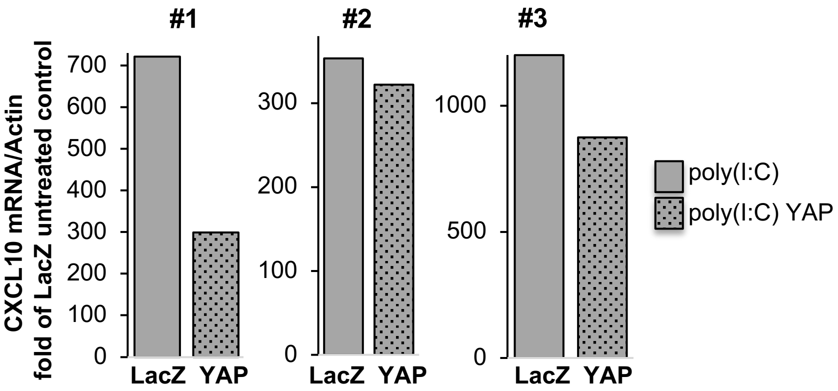

Figure S8. Intra-batch variation of YAP overexpression on Poly(I:C)-induced CXCL10 expression in different Islet donors. Human islets were transduced with Ad-YAP or Ad-LacZ (control) and subsequently transfected with Poly(I:C) for 24 hours. qPCR analysis of *CXCL10* mRNA expression reveals large variation in the inflammatory gene response to Poly(I:C) among three human islet donors. This figure presents additional data analyses related to Figure 7C. n=1 for each bar.

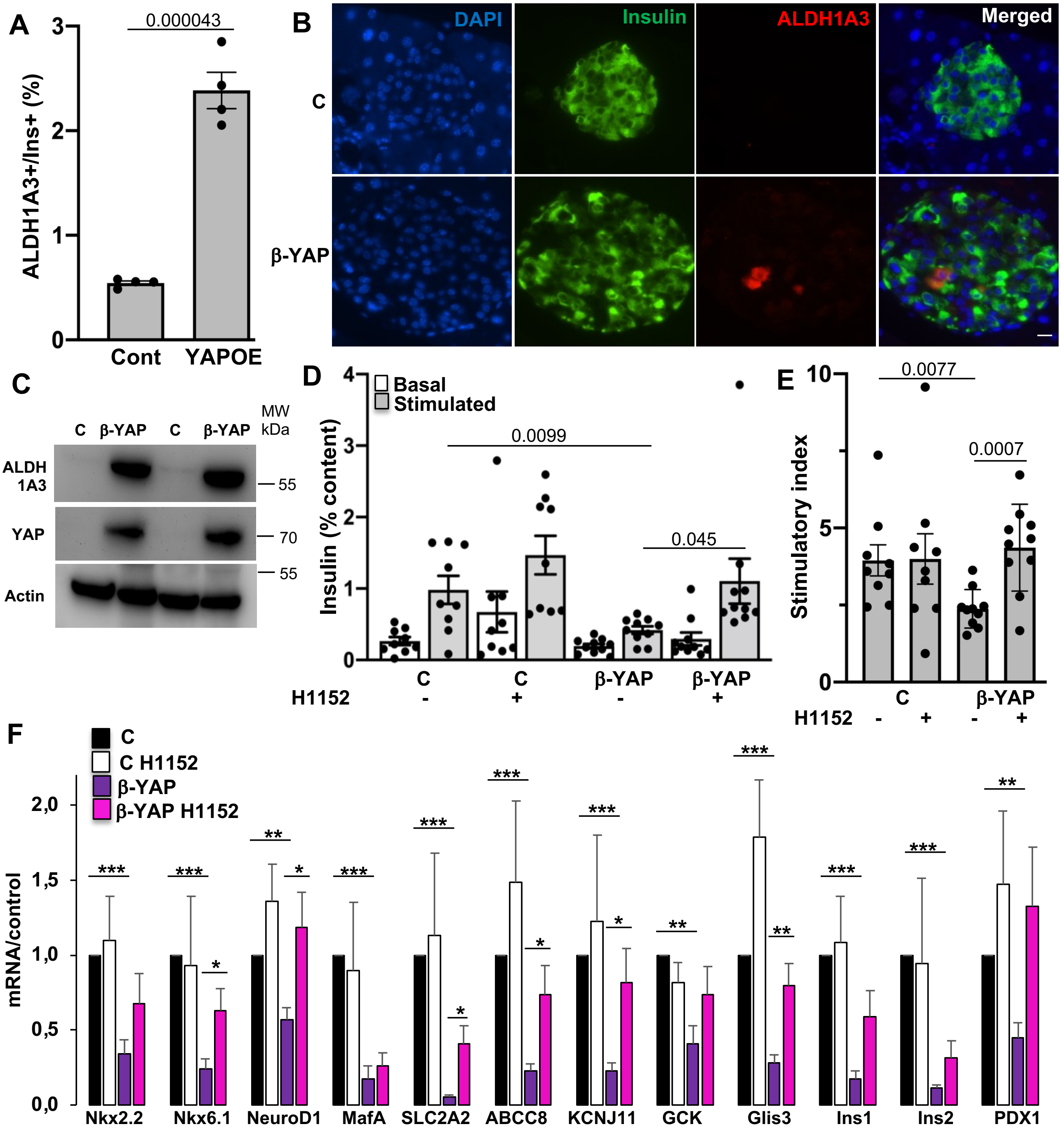

Figure S9. YAP induces β-cell dedifferentiation. YAP was transiently induced by doxycycline administration in drinking water for 2 weeks (β-YAP) and results compared to -DOX/-YAP (control; C). **(A,B)** Quantitative analyses **(A)** and representative images **(B)** from triple stainings for ALDH1A3, insulin and DAPI expressed as percentage of ALDH1A3-positive β-cells (n=4). **(C)** Western blot of ALDH1A3 and YAP in mouse islets (n=2). **(D-F)** Isolated mouse islets from β-YAP mice and respective control were left untreated or treated with 10µM H1152 for 48 hours. **(D)** Insulin secretion during 1h-incubation with 2.8 mM (basal) and 16.7 mM glucose (stimulated), normalized to insulin content and **(E)** stimulatory index denotes the ratio of stimulated to basal insulin secretion (n=9-10). **(F)** qPCR analysis of *MafA, Nkx6.1, Slc2a2, NeuroD1, GCK, Ins1, Ins2, Pdx1, Nkx2.2, Glis3*, *Abcc8, and Kcnj11* (n=5-10). Data are expressed as means ± SEM. *P*-values were calculated by two-tailed unpaired Student *t*-test. *p<0.05, **p<0.01, ***p<0.001. Scale bar depicts 10µm.

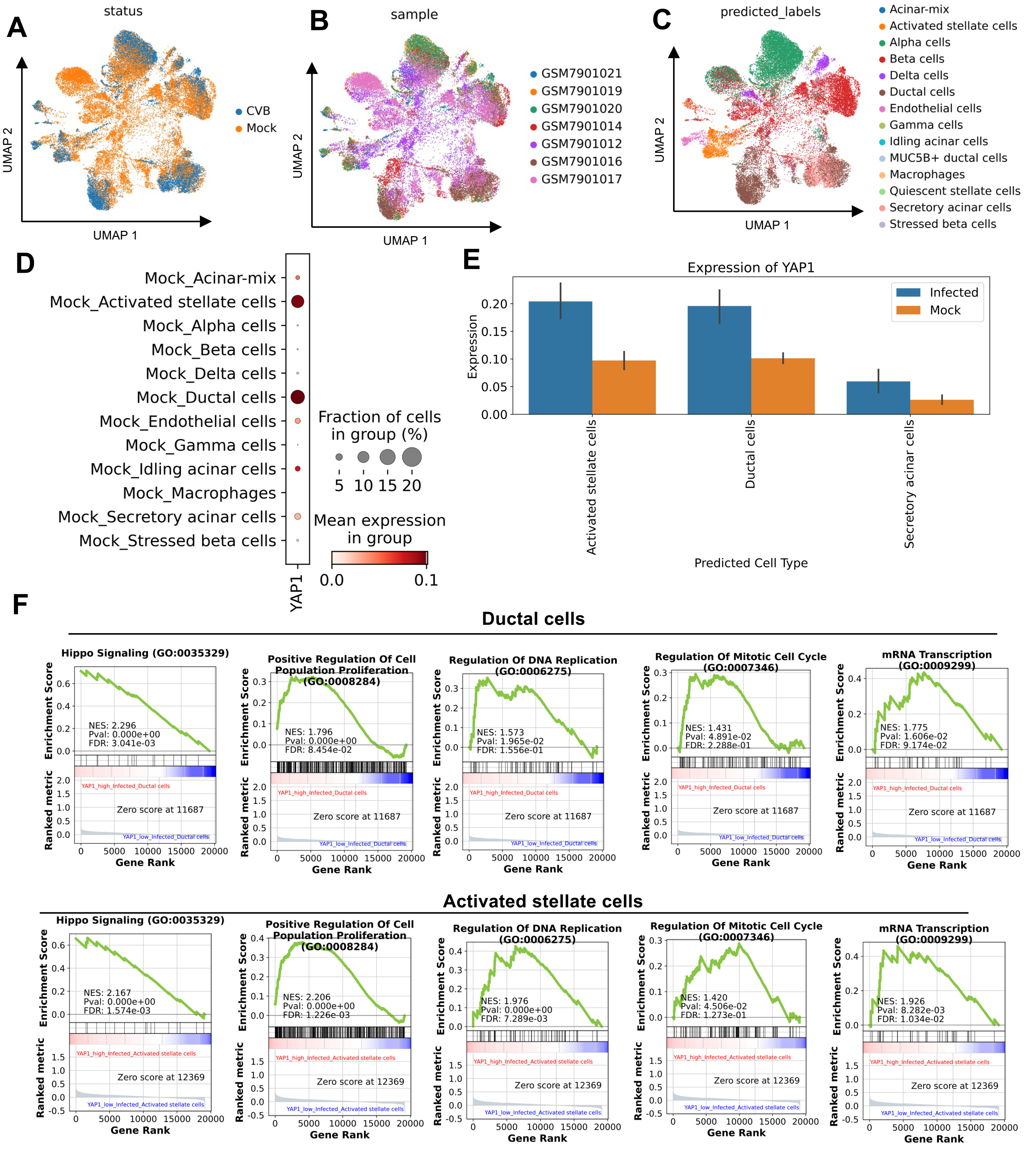

Figure S10. Reanalysis of scRNA-seq data from Mock and CVB4-infected human islets/exocrine cells. (A) UMAP visualization of cells colored by infection status (Mock vs. CVB4-infected). **(B)** UMAP visualization of corresponding donor identities. **(C)** UMAP representation of cell types classified using CellTypist. **(D)** Dot plot showing *Yap1* expression across cell types in the Mock condition. **(E)** Bar plot comparing *Yap1* expression levels in activated stellate, ductal, and secretory acinar cells between Mock and CVB4-infected conditions. **(F)** GSEA visualization of cellular landscape cascades in ductal and activated stellate cells, comparing "YAP1-high" vs. "YAP1-low" subpopulations of infected cells. GSEA was performed using the GSEAPY tool with the GO Biological Process 2023 gene sets, applying a Kolmogorov-Smirnov-like enrichment score. Statistical significance was assessed via permutation testing followed by false discovery rate (FDR) correction.

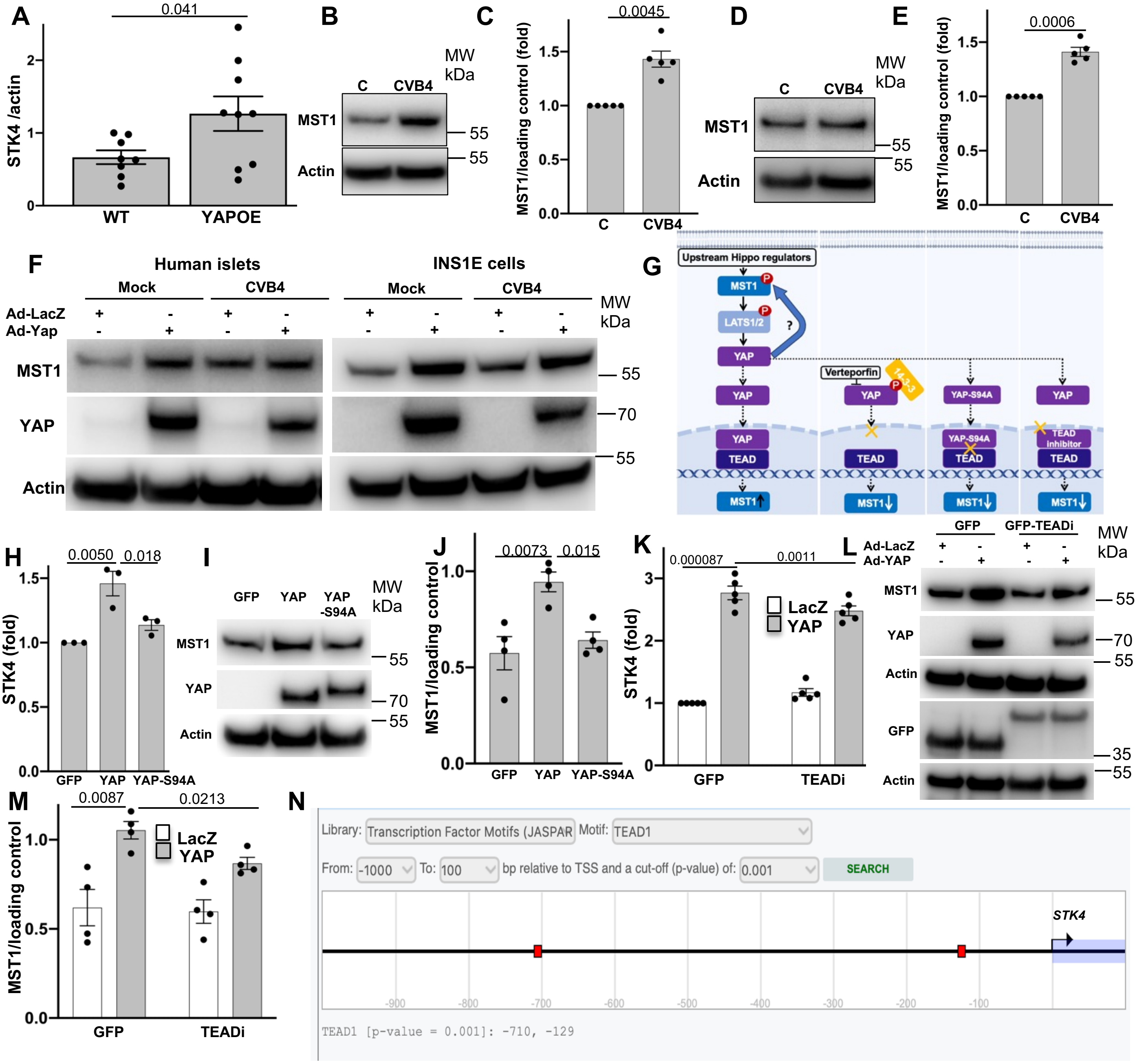

Figure S11. YAP/TEAD controls MST1 expression. (A) qPCR for *STK4* mRNA expression in islets isolated from β-YAP and control mice recovered after isolation overnight (n=8-9 mice). **(B,C,F)** INS-1E cells and **(D,E,F)** human islets infected with CVB4 (MOI=5) for 24h (INS-1E) or CVB4 (MOI=10) for 48h (human islets). **(B-E)** Representative Western blot and pooled densitometry analysis of MST1 in INS-1E cells (**B,C**; n=5) and human islets (**D,E**; n=5), **(F;** n=3). **(G)** Schematic presentation of complementary approaches to block YAP/TEAD signaling. **(H-J)** INS-1E cells transfected with GFP, active YAP or YAP-S94A constructs for 48h. **(H)** qPCR for *STK4* mRNA expression in INS-1E cells (n=3). **(I)** Representative Western blot and **(J)** pooled quantitative densitometry analysis of MST1 in INS-1E cells (n=4). **(K-M)** INS-1E cells transfected with GFP, or TEADi constructs and then transduced with Ad-YAP or Ad-LacZ control for 48h. **(K)** qPCR for *STK4* mRNA expression in INS-1E cells (n=5). **(L)** Representative Western blot and **(M)** pooled quantitative densitometry analysis of MST1 in INS-1E cells (n=4). **(N)** Predicted TEAD1 binding sites in the rat STK4 promoter identified by EPD (https://epd.epfl.ch/index.php). Data are expressed as means ± SEM. *P*-values were calculated by two-tailed unpaired Student *t*-test for A,M and by two-tailed paired Student *t*-test for C,E,K and by one-way ANOVA with Holm-Sidak multiple comparisons correction for H and J.

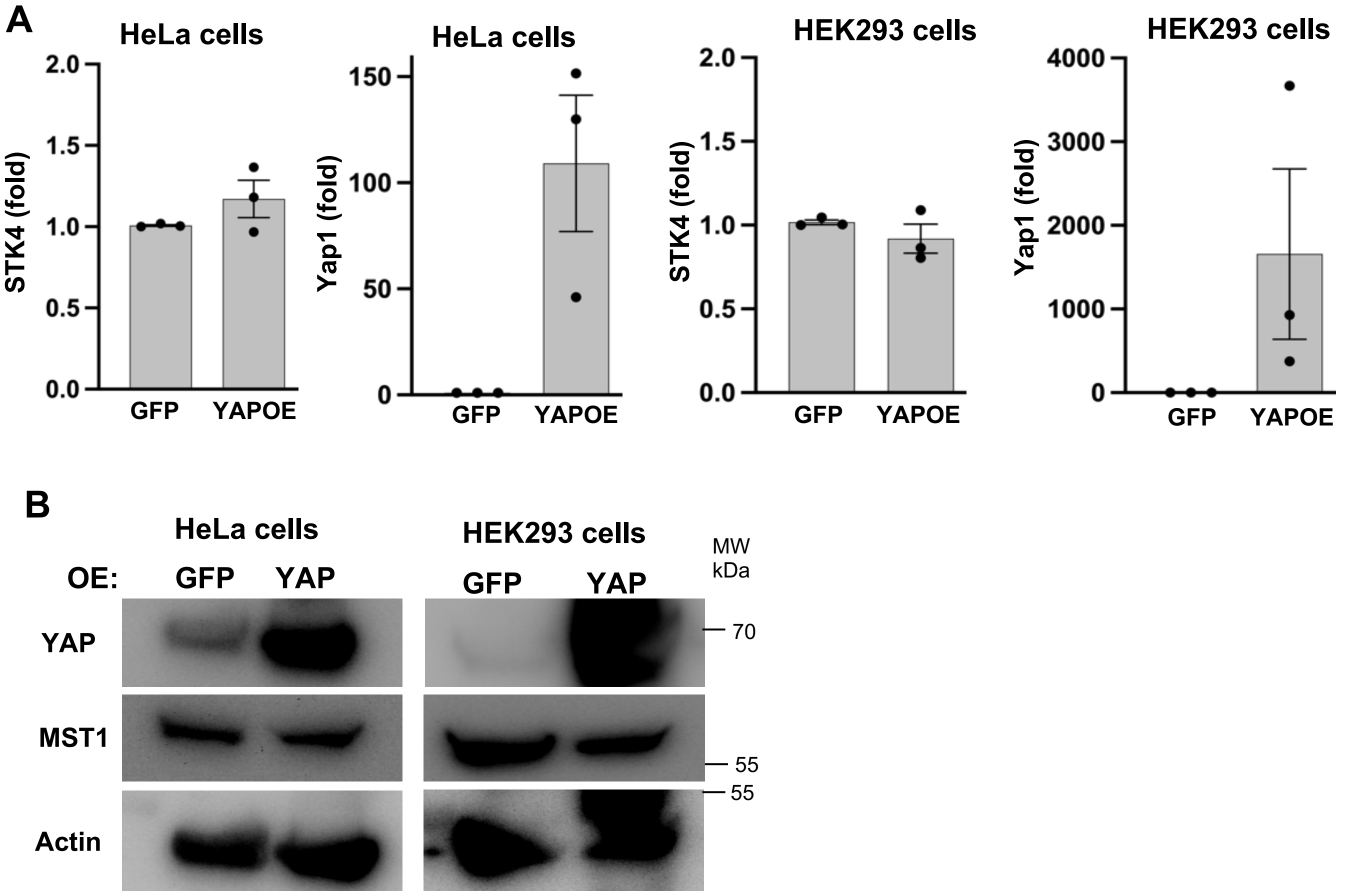

Figure S12. YAP overexpression does not induce STK4/MST1 expression. HeLa or HEK293 cells transfected with GFP control or activated YAP (YAP-S127A) constructs for 24h. **(A)** qPCR for *Yap1* or *STK4* (gene encoding MST1) mRNA expression (n=3 independent experiments). **(B)** Representative Western blot of YAP and MST1 proteins (n=3 independent experiments). Data are expressed as means ± SEM.

#	ID	RRID: SAMN	type	Aab	Age	Diab. Durat	gender	Ethnicity	BMI	HbA1c	C-pep (ng/ml)
**1**	6375	15879428	control		≥ 20	0	male	White	32	5.7	17.3
**2**	6413	15879466	control		≥10	0	female	White	19	5.6	5.3
**3**	6278	15879332	control		≥10	0	female	African American	21	6.3	4.5
**4**	6384	15879437	control		≥10	0	male	White	18	4.8	0.7
**5**	6254	15879310	control		≥20	0	male	White	31	5.3	6.4
**6**	6406	15879459	control		<10	0	male	White	17	5.1	4.1
**7**	6401	15879454	control		≥20	0	female	Hispanic	31	5.8	12.8
**8**	6112	15879169	control		<10	0	female	Hispanic	18	5.6	5.1
**9**	6516	18053200	control		≥20	0	male	White	29	5.5	8.9
**10**	6539	25652250	control		≥20	0	male	Hispanic	19	5.7	39.2
**11**	6544	25652255	control		≥10	0	male	African American	23	5.2	8.7
**12**	6530	18053212	control		≥10	0	female	African American	23	6	7.4
**13**	6525	18053208	control		≥10	0	male	Hispanic	19	5.4	2.7
**14**	6339	15879393			≥20	0	male	White	25	5.3	10.6
M					**18**				**23**	**6**	**9.5**
**1**	6421	15879474	Aab+	GADA+	<10	0	male	Hispanic	18	5.6	1.8
**2**	6400	15879453	Aab+	GADA+	≥20	0	male	Hispanic	22	5.5	4.2
**3**	6429	15879482	Aab++	GADA+mIAA+	≥20	0	male	African American	20	5.5	2.3
**4**	6424	15879477	Aab++	GADA+mIAA+	≥10	0	male	White	51	5.8	7.0
**5**	6397	15879450	Aab+	GADA+	≥20	0	female	White	30	6	12.8
**6**	6303	15879357	Aab+	GADA+	≥20	0	male	White n	32	5.4	3.0
**7**	6388	15879441	Aab++	GADA+mIAA+	≥20	0	female	Hispanic	26	5.7	1.4
**8**	6310	15879364	Aab+	GADA+	≥20	0	female	Hispanic	22		10.5
**9**	6347	15879401	Aab+	mIAA+	<10	0	male	White	20		3.3
**10**	6090	15879147	Aab+	GADA+	<10	0	male	Hispanic	19		5.3
**11**	6558	30386847	Aab+	GADA+			female	African American	28	4.4	8.0
**12**	6532	18053214	Aab+	GADA+	≥20	0	male	Hispanic	24	5.9	22.1
**13**	6553	30386843	Aab+	mIAA+	≥10	0	female	Hispanic	25	8.4	4.6
**14**	6521	18053204	Aab+++	GADA+ IA-2A+ ZnT8A+	≥20	0	male	Hispanic	24	5.8	7.4
**15**	6517	18053201	Aab+	GADA+	≥20	0	male	White	27	6	9.7
**16**	6301	15879355	Aab+	GADA+	≥20	0	female	African American	32	5.5	3.9
M					**19**				**26**	**6**	**6.7**
**1**	6299	15879353	T1D	mIAA	≥20	23	male	White	31.8		<0.05
**2**	6367	15879420	T1D	-	≥20	2	male	White	25.7	8.8	0.39
**3**	6396	15879449	T1D	-	≥10	2	female	White	22.6	13.4	0.06
**4**	6380	15879433	T1D	-	≥10	0	female	African American	14.6	13.5	0.22
**5**	6371	15879424	T1D	GADA+ IA-2A+ mIAA+ ZnT8A+	≥10	2	female	White	16.6	9.5	0.11
**6**	6211	15879267	T1D	GADA+ IA-2A+ ZnT8A+ mIAA+	≥20	4	female	African American	24.4	10.5	<0.05
**7**	6414	15879467	T1D	GADA+ mIAA+ZnT8A+	≥20	0.43	male	African American	28.4	14	0.16
**8**	6405	15879458	T1D	GADA+ IA-2A+ ZnT8A+	≥20	0.6	female	Hispanic	42.5	7	1.84
**9**	6046	15879103	T1D	IA-2A+ ZnT8A+	≥10	8	female	White	25.2		<0.05
**10**	6362	15879415	T1D	GADA+	≥20	0	male	White	28.5	10	0.38
**11**	6563	30386851	T1D	IA2A+	≥10	0	female	White	25.5	9.6	1.04
**12**	6536	18242780	T1D	GADA+	≥20	4	female	White	25.4	12.7	0.04
**13**	6523	18053206	T1D	GADA+ mIAA+	≥10	3	female	African American	22.5	11.1	0.04
**14**	6550	25652261	T1D	GADA+ ZnT8A+	≥20	0	male	White	16.4	14	<0.02
**15**	6526	18053209	T1D	IA-2A+ mIAA+	≥20	1	male	Hispanic	23	6.6	0.07
M					**21**	**3**			**25**	**11**	**0.3**

Suppl.Table 1. nPOD inventory donor case IDs and demographics.
