# Supplementary material for: The Hippo terminal effector YAP boosts enterovirus replication in type 1 diabetes
Source: Nat Commun. 2025 Oct 6;16:8882. doi: 10.1038/s41467-025-64508-6 (PMC12500894; doi:10.1038/s41467-025-64508-6)
Supplement: Supplementary file 2 — Reporting Summary [file 41467_2025_64508_MOESM2_ESM.pdf]

nature portfolio

Corresponding author(s): Amin Ardestani, Kathrin Maedler

Last updated by author(s): 23.08.2025

Reporting Summary

Nature Portfolio wishes to improve the reproducibility of the work that we publish. This form provides structure for consistency and transparency in reporting. For further information on Nature Portfolio policies, see our Editorial Policies and the Editorial Policy Checklist.

Statistics

For all statistical analyses, confirm that the following items are present in the figure legend, table legend, main text, or Methods section.

n/a	Confirmed	
☐	☒	The exact sample size (*n*) for each experimental group/condition, given as a discrete number and unit of measurement
☐	☒	A statement on whether measurements were taken from distinct samples or whether the same sample was measured repeatedly
☐	☒	The statistical test(s) used AND whether they are one- or two-sided<br>*Only common tests should be described solely by name; describe more complex techniques in the Methods section.*
☒	☐	A description of all covariates tested
☐	☒	A description of any assumptions or corrections, such as tests of normality and adjustment for multiple comparisons
☐	☒	A full description of the statistical parameters including central tendency (e.g. means) or other basic estimates (e.g. regression coefficient) AND variation (e.g. standard deviation) or associated estimates of uncertainty (e.g. confidence intervals)
☐	☒	For null hypothesis testing, the test statistic (e.g. *F*, *t*, *r*) with confidence intervals, effect sizes, degrees of freedom and *P* value noted<br>*Give P values as exact values whenever suitable.*
☒	☐	For Bayesian analysis, information on the choice of priors and Markov chain Monte Carlo settings
☒	☐	For hierarchical and complex designs, identification of the appropriate level for tests and full reporting of outcomes
☒	☐	Estimates of effect sizes (e.g. Cohen's *d*, Pearson's *r*), indicating how they were calculated

Our web collection on statistics for biologists contains articles on many of the points above.

Software and code

Policy information about availability of computer code

Data collection	Immunostaining and imaging were performed using a Nikon MEA53200 microscope with NIS-Elements software or an inverse Nikon Ti2-A MEA54100 microscope (NIKON GmbH, Düsseldorf, Germany) with NIS-Elements BR-ML software. Images were acquired at different magnifications depending on the experiment. Western blot analysis was conducted using DocIT®LS image acquisition 6.6a (UVP). qPCR experiments were performed using the Applied Biosystems ViiA 7 real-time PCR system.
Data analysis	Immunostaining images were analyzed and quantified using NIS-Elements software (Nikon, version 3.22.11) and ImageJ.JS (v0.5.6). YAP-positive areas in the exocrine pancreas were quantified as a percentage of the total tissue area. The fraction of YAP-positive cells in pancreatic islets was determined by manually counting YAP-positive cells, normalized to the total number of chromogranin-positive cells. Western blots were quantified using DocIT®LS image acquisition 6.6a (UVP). qPCR data were analyzed using the Applied Biosystems ViiA 7 real-time PCR system, with gene expression normalized to housekeeping controls. For infection analysis, the percentage of VP1-positive cells was calculated by counting infected cells and normalizing to the total number of DAPI-stained nuclei. In RNAScope analysis, the percentage of YAP-mRNA+ cells was quantified relative to the total number of nuclei, while CTGF RNA puncta were assessed per nucleus. Apoptosis and infection in human islets were quantified as the percentage of TUNEL/insulin or VP1/insulin double-positive cells, normalized to the total insulin-positive cells per islet.

For manuscripts utilizing custom algorithms or software that are central to the research but not yet described in published literature, software must be made available to editors and reviewers. We strongly encourage code deposition in a community repository (e.g. GitHub). See the Nature Portfolio guidelines for submitting code & software for further information.

Data

Policy information about availability of data

All manuscripts must include a data availability statement. This statement should provide the following information, where applicable:

- Accession codes, unique identifiers, or web links for publicly available datasets
- A description of any restrictions on data availability
- For clinical datasets or third party data, please ensure that the statement adheres to our policy

All data generated or analyzed during this study are included in this article and its supplementary information files. All original source data are provided in the Source Data file.

Research involving human participants, their data, or biological material

Policy information about studies with human participants or human data. See also policy information about sex, gender (identity/presentation), and sexual orientation and race, ethnicity and racism.

Reporting on sex and gender	Cells (human islets) and tissues (pancreatic sections) from both male and female organ donors were used. Formalin-fixed paraffin-embedded (FFPE) pancreatic tissue sections were obtained from well-characterized organ donors through the Network for Pancreatic Organ Donors with Diabetes (nPOD).
Reporting on race, ethnicity, or other socially relevant groupings	Race and ethnicity for formalin-fixed paraffin-embedded (FFPE) pancreatic tissue sections obtained from human organ donors reported in Table S1.
Population characteristics	Human islet isolations from male and female organ donors with reported HbA1c <6 were used in this study. Pancreas sections from organ donors with HbA1c ≤6 (controls; Aab+) and reported T1D diagnosis were used in this study.
Recruitment	Human islets were isolated from pancreases of nondiabetic organ donors (both male and female) at University of Lille, Strasbourg and ProdoLabs. France has presumed consent legislation in place for deceased donors. Human islet preparations insufficient in number for clinical transplantation were used for research when consent for research donation has been given. Single pancreas sections were distributed through the nPOD tissue collection of well-preserved pancreases from organ donors.
Ethics oversight	Ethical Committee University of Bremen, Comité d'Ethique du Centre Hospitialier et Universitaire de Lille, Islet for Basic Research program; European Consortium for Islet Transplantation ECIT and Integrated Islet Distribution Program IIDP. Ethical approval for the use of formalin-fixed paraffin-embedded (FFPE) pancreatic tissue sections obtained from well-characterized organ donors from the network for Pancreatic Organ Donors with diabetes (nPOD) had been granted by the Ethics Committee of the University of Bremen.

Note that full information on the approval of the study protocol must also be provided in the manuscript.

Field-specific reporting

Please select the one below that is the best fit for your research. If you are not sure, read the appropriate sections before making your selection.

☒ Life sciences ☐ Behavioural & social sciences ☐ Ecological, evolutionary & environmental sciences

For a reference copy of the document with all sections, see nature.com/documents/nr-reporting-summary-flat.pdf

Life sciences study design

All studies must disclose on these points even when the disclosure is negative.

Sample size	For all of experiments in mouse and human islets and cell lines, samples were collected from at least 3 biologically independent experiments or biologically independent samples unless otherwise stated. These were chosen based on availability and our previous studies without using statistical methods for sample size determination. All findings were confirmed in a variety of biological models and by several techniques. For confirmatory experiments on restricted material, i.e. using human islets, at least 2 independent experiments from 2 independent organ donors were used. The exact values of n (refers to number of donors or mice, or number of independent biological experiments or independent measurements/positions), and statistical significance are reported in the figure legends.
Data exclusions	No data were excluded from the analyses.
Replication	All attempts for replication were successful by different co-authors of this study. For all essential experiments in mouse and human islets and cell lines, 3-5 independent experiments were performed (unless otherwise stated), for all confirmatory replicative experiments in an additional biological model, at least 2 independent experiments were performed.
Randomization	All cell culture experiments were performed in individual dishes or tissue culture plates, allowing for random sample assignment to different treatment conditions after cell seeding. After genotyping and confirmation of β-YAP-OE status, animals were assigned a unique ID. Animals were then randomly allocated to cages, with groups receiving either doxycycline (β-YAP-OE) or no doxycycline (respective control).
Blinding	Most of histological analyses including YAP staining, apoptosis, viral replication, and infection were done fully blinded. Labeling of sections

was done by numbering at the time of sectioning. Western blot loading of samples could not have done in a blinded fashion because we want the control condition and respective treatment conditions run in subsequent lanes to allow comparisons. Mouse cages had to be assigned and labelled thereafter due to strict animal housing conditions and daily documentations of different mice of various genotypes and treatment conditions.

Reporting for specific materials, systems and methods

We require information from authors about some types of materials, experimental systems and methods used in many studies. Here, indicate whether each material, system or method listed is relevant to your study. If you are not sure if a list item applies to your research, read the appropriate section before selecting a response.

Materials & experimental systems

n/a	Involved in the study
☐	☒ Antibodies
☐	☒ Eukaryotic cell lines
☒	☐ Palaeontology and archaeology
☐	☒ Animals and other organisms
☒	☐ Clinical data
☒	☐ Dual use research of concern
☒	☐ Plants

Methods

n/a	Involved in the study
☒	☐ ChIP-seq
☒	☐ Flow cytometry
☒	☐ MRI-based neuroimaging

Antibodies

Antibodies used

Western Blot analysis: rabbit anti-cleaved caspase-3 (#9664, CST), rabbit anti-Total YAP (#14074, clone D8H1X, CST), rabbit anti-active YAP (#ab223126, clone EPR19812, abcam), rabbit anti-Phospho-YAP (Ser127) (#4911, CST), rabbit anti-MST1 (#3682, CST), rabbit anti-ALDH1A3 (#NBP2-15339), rabbit anti-GAPDH (#2118, CST), rabbit anti-β-actin (#4967, CST), and mouse anti-Enterovirus/VP1 (clone 5-D8/1 #M7064, Dako). All primary antibodies were used at 1:1,000 dilution in 1xTris-buffered saline plus Tween-20 (1xTBS-T) containing 5% BSA and 0.5% NaN3. Later, membranes were incubated with horseradish-peroxidase-linked anti-rabbit or anti-mouse secondary antibodies (Jackson ImmunoResearch, PA, USA) and developed using Immobilon Western chemiluminescence assay system (Millipore, MA, USA).
Stainings: guinea pig anti-insulin (#IR002, FLEX polyclonal DAKO), mouse, mouse anti-Enterovirus/VP1 (clone 5-D8/1 #M7064, Dako), mouse anti-chromogranin (#ab715, Abcam), mouse anti-cytokeratin 19/CK-19 (#15463-1, Abcam), rabbit anti-ALDH1A3 (#NBP2-15339), rabbit anti-Total YAP (#14074, clone D8H1X, CST), and rabbit anti-active YAP (#ab223126, clone EPR19812, abcam). The next day sections were incubated with Cy3-conjugated donkey anti-mouse (#715-165-150), FITC-conjugated donkey anti-guinea pig (706-096-148) or FITC- conjugated donkey anti-mouse (#715-095-150) secondary antibodies (all from Jackson Immuno Research Laboratories, West Grove, PA; 1:100 dilution) for 1h at RT or 37°C.
YAP immunohistochemistry: rabbit anti-YAP (D8H1X, #14074, CST) antibody alone or in combination with mouse anti-chromogranin (#ab715, Abcam) antibody overnight. A day after, sections were washed with PBS and were incubated with rabbit poly-HRP-conjugated secondary antibody for 1h at RT. To amplify the signal, a Tyramide working solution was prepared according to the manufacturer's instructions by adding the Tyramide solution and hydrogen peroxide into the reaction buffer.

Validation

All antibodies were validated for their expression on mouse and human islet or pancreatic sections and used according to the manufacturer, who has validated the antibodies before. Pos. and neg. controls (siRNA and overexpression conditions) were included to validate the Ab for the use in our specific models whenever available. All antibodies have been validated and reported in prior studies.

Eukaryotic cell lines

Policy information about cell lines and Sex and Gender in Research

Cell line source(s)

The clonal rat β-cell line INS-1E was kindly provided by Claes Wollheim (Geneva & Lund University). The immortalized cell line Hela and HEK293 was purchased from American Type Culture Collection (ATCC, Manassas, VA, USA). The human pancreatic exocrine ductal cell line PANC-1 was generously provided by Cenap Güngör, Universitätsklinikum Hamburg-Eppendorf (UKE). CVB-permissive FKRH4 cells were generously provided by Andreas Dotzauer, University of Bremen.

Authentication

INS-1E cells secreted insulin and all tests for beta-cell specific transcription factors were positive, as tested and reported earlier by us (Oncotarget. 2016 Aug 2;7(31):48963-48977; Nat Med. 2014 Apr;20(4):385-397). Further authentications were not performed.

Mycoplasma contamination

We routinely test all cell lines for mycoplasma. Tests were negative.

Commonly misidentified lines
(See ICLAC register)

No commonly misidentified lines were used.

Animals and other research organisms

Policy information about studies involving animals; ARRIVE guidelines recommended for reporting animal research, and Sex and Gender in Research

Laboratory animals	β-cell-specific YAP overexpressing (YAP-OE) mice were generated by crossing inducible active YAP overexpressing mice (TetO-YAPSer127A, provided to our lab in collaboration with Fernando Camargo, Boston Children's Hospital, Boston, MA) with mice carrying the tetracycline transactivator (tTA) under the control of the insulin promoter (RIP-rtTA mice, kindly provided by Al Powers, Vanderbilt University Medical Center, Nashville, TN, USA). Background is C57Bl/6. In the Rip-Ins2-TetO-hYAP1-S127A mice, rtTA gene becomes activated specifically in the islet β-cells due to the Ins2 promoter. Upon doxycycline (a tetracycline analog) treatment, the rtTA protein in these cells can bind to the tet-response element (TRE) and subsequently causing the transcription of the constitutively active form of YAP gene which is under a CMV promoter element. This system enables a fine-tuned spatio-temporal control over the expression of the aYAP gene in the pancreatic β-cells. NOD/ShiLtJ Strain #:001976 RRID:IMSR_JAX:001976; Common Name: NOD were bred and ordered from JAX, background Jcl:ICR.
Wild animals	N/A
Reporting on sex	Both male and female mice were used. Following isolation, in vitro cultures were performed on islets isolated from both male and female βYAP-OE and respective control mice.
Field-collected samples	All mice used in this experiment were housed in a temperature-controlled room with a 12-h light-dark cycle and were allowed free access to food and water in agreement with NIH animal care guidelines, §8 German animal protection law, German animal welfare legislation and with the guidelines of the Society of Laboratory Animals (GV-SOLAS) and the Federation of Laboratory Animal Science Associations (FELASA) or with protocols approved by the Animal Care Committee of the University of British Columbia (NOD mice).
Ethics oversight	All protocols were approved by the Bremen Senate (Senator for Science, Health and consumer protection) or with protocols approved by the Animal Care Committee of the University of British Columbia (NOD mice) and we have complied with all relevant ethical regulations for animal testing and research.

Note that full information on the approval of the study protocol must also be provided in the manuscript.
